# MicroRNA-mediated regulatory mechanisms in cow’s ileum and ileum lymph node in response to *Mycobacterium avium* subspecies *paratuberculosis* infection

**DOI:** 10.1038/s41598-025-23969-x

**Published:** 2025-12-03

**Authors:** Mengqi Wang, Nathalie Bissonnette, Pier-Luc Dudemaine, Eveline M. Ibeagha-Awemu

**Affiliations:** Sherbrooke Research and Development Centre, Agriculture and Agri-Food Canada, Sherbrooke, QC Canada

**Keywords:** Johne’s disease, MiRNA, Immune response, Ileum, Ileum lymph node, Liver, Genetics, Molecular biology, Diseases

## Abstract

*Mycobacterium avium* subsp. *paratuberculosis* (MAP) causes Johne’s disease (JD), a chronic enteritis infection affecting ruminants with significant economic implications for the dairy industry. The molecular regulatory mechanisms governing host responses to MAP infection remain unclear. This study elucidated the roles of microRNAs (miRNAs) in the ileum (IL), ileal lymph node (ILLN), and liver of cows during subclinical MAP infection. Tissues (IL, ILLN, and liver) were collected from four JD-positive (JDP), five MAP-tolerant (MAPT), and five healthy control (HC) cows. MiRNA transcriptomes were analyzed using miRNA-sequencing, differential miRNA expression, correlation, and functional analyses. A total of 441, 405, and 201 miRNAs were identified in IL, ILLN, and liver, respectively, including 12 miRNAs highly expressed across all three tissues. ILLN exhibited more differentially expressed (DE) miRNAs, including 20 (JDP vs. HC), 21 (MAPT vs. HC), and 15 (JDP vs. MAPT) than the IL: 17 (JDP vs. HC), 8 (MAPT vs. HC), and 6 (JDP vs. MAPT). MiRNAs DE in both IL and ILLN (bta-miR-146b, bta-miR-375, bta-miR-21-5p, bta-miR-146a, bta-miR-125a, bta-miR-100, bta-miR-147, bta-miR-99a-5p, bta-miR-320 from JDP vs. HC, and bta-miR-146a, bta-let-7b from MAPT vs. HC comparisons) have roles in the immune process suggesting their potential impacts on the host response to MAP infection. The target genes of IL DE miRNAs were overrepresented in more gene ontology terms (68) while those of the ILLN were overrepresented in more pathways (20) demonstrating tissue specificity. In addition, the majority of DE miRNAs and overrepresented functional terms were unique to comparison groups and phenotypes while few were shared by two or more comparison groups and phenotype or infection status. Most of the functional terms such as cytokine receptor activity, regulation of leukocyte mediated immunity, T cell receptor signaling pathway, positive regulation of interleukin-4 production among others, have immune related functions revealing important roles of DE miRNAs in the host immune response to MAP infection. In summary, these findings underscore the pivotal regulatory roles of the identified miRNAs in shaping the host response of cows to JD. Moreover, the results indicated that mostly different sets of miRNAs, biological process and pathways were driving the JDP and MAPT phenotypes, as well as emphasize the tissue-specific nature of the miRNA response to JD, shedding light on the nuanced intricacies of host-pathogen interactions in response to MAP infection.

## Introduction

Johne’s disease (JD), caused by *Mycobacterium avium* subsp. *paratuberculosis* (MAP), is a chronic progressive disease causing inflammatory disorder of the intestine and other organs of ruminants (cattle, sheep and goats)^1,2^ and other non-ruminant species such as pig, rabbit, and horse^3–5^. For the dairy industry, JD causes huge economic losses related to low milk yield, fertility problems, high culling rate, loss of body weight, the spread of MAP to other herd mates and environmental contamination^6–8^.

MAP infects the gastrointestinal tract of cattle during the first 6 months of life, sometimes starting in utero, and most infected cattle show clinical signs within 2 to 6 years^9,10^. The disease is generally classified as (i) subclinical infection, which is defined as *MAP* infection without demonstrable pathology in tissues; (ii) subclinical disease, which is defined as the presence of pathology in tissues without weight loss or diarrhea; and (iii) clinical disease, which is defined as the presence of pathology and weight loss and/or diarrhea^11^. The risk of disease transmission to other herd mates and environmental contamination is high during the subclinical phase of the infection. While the identification and isolation of infected animals during the early stages of the disease is an efficient way to control the transmission of the pathogen, current detection methods (e.g. ELISA test, fecal PCR or fecal culture) are not reliable or have low sensitivity^12–14^. For example, blood ELISA sensitivity (< 30%) is too low to be reliable for detecting subclinical MAP-infected cows^15–19^, while sensitivity of fecal culture method is low for subclinical cows^20,21^. Therefore, the current lack of effective treatment of MAP-infected cows and sensitive methods to detect MAP infection justifies the search for biomarkers associated with the subclinical stage of disease progression.

Being a complex disease, deeper understanding of its genetic basis and molecular mechanisms will support the development of more effective monitoring and control measures. Genome-wide and candidate gene association studies have associated genomic variations such as single nucleotide polymorphisms (SNPs) and copy number variants in some immune response genes and genomic regions with susceptibility^22–29^ and resistance^30–33^ to MAP infection, which could be used in genomic selection to reduce cattle susceptibility/resistance and economic losses. Furthermore, different perspectives on the immunopathologic response to MAP infection have been explored by transcriptional changes in MAP infected Peyer patches^34^; blood^35–37,38^), ileocecal valve^35,39,40^, macrophages^41–44^, jejunum and ileum^45,46^, jejunum and ileum or ileal lymph nodes^45^ and salivary glands^47^. Furthermore, differentially expressed genes from these studies associated immune system-related pathways, such as Leukocyte transendothelial migration, T/B-cell receptor signaling pathways, Chemokine signaling pathway, Toll-like receptor signaling pathway, Cytokine-cytokine receptor interaction, NF-kB signaling and NOD-like receptor pathway, among others, with MAP infection in cattle^39,41,44,45^. These data suggest the importance of further research into the molecular mechanisms underlying MAP infection, which could uncover new and useful biomarkers for the control of JD. Moreover, the potential association of JD with human Crohn’s disease and other human diseases has attracted more research attention to JD^48–50^.

In bovine, noncoding RNAs including microRNAs (miRNAs) and long noncoding RNAs have been associated to many biological processes and diseases, including JD^50–53^. In particular, altered microRNAs (miRNA) expressions have been identified in intestinal tissues^40,54^, feces^55^, sera^56–58^, blood^37,54^, macrophages^59,60^, mammary epithelial cells^61^, and salivary glands and jejunum tissue^46^ from MAP infected cows. These studies revealed distinct miRNA profiles in MAP-infected cattle compared to uninfected individuals, as well as their involvement in immune regulatory pathways and biological processes, thereby emphasizing broad roles for miRNAs in the post-transcriptional regulation of mechanisms altered during MAP infection. Moreover, miRNAs have been noted to play roles during early life when calves have higher susceptibility to MAP infection^40,62–64^.

The small intestine and specially the ileum is the main target organ or entry point of MAP. While in the small intestine, MAP translocate the intestinal barrier via M-cells and enterocytes^65^. Through M-cells, MAP enters the ileal Peyer’s patches causing infection in macrophages and eventually upon release migrate to lymph nodes and other intestinal cells or shed through feces^65–68^. The draining mesenteric lymph nodes are therefore an essential element of defense against infectious agents including MAP. The molecular mechanisms underlying MAP infection in subclinical cows are not well known. The miRNA transcriptome analysis of infected gastrointestinal tract tissues will enhance understanding of MAP pathogenesis and the development of control measures. In this study, therefore, whole miRNA transcriptome was profiled for ileum (IL), ileum or ileal lymph node (ILLN) and liver tissues from healthy cows, cows with JD and MAP tolerant cows, and further investigated to unravel the potential functions of miRNAs in the pathogenesis to JD.

## Materials and methods

### Animal selection and Johne’s disease diagnosis

Animal sampling was performed following the guidelines for institutional animal use by the Canadian Council on Animal Care, and experimental procedures and ethical approval for the research was granted by the Animal Care and Ethics Committee of Agriculture and Agri-Food Canada (AAFC) (No. 466).

Animals for this study were selected from commercial dairy farms with a history of MAP infection status in the provinces of Quebec and Ontario, Canada. They were collected two times per year (every 5–7 months) by technicians or animal handlers under the supervision of a veterinarian and AAFC, as previously described^53^. Procedures for collecting blood and feces and testing for the presence of MAP antibodies using Pourquier ELISA assay (IDEXX Laboratories, Markham, Ontario, Canada) and quantification of MAP fecal excretion by qPCR (VetMAX™ Golf MAP Detection Kit, Life Technologies Inc., Burlington, Ontario, Canada) have been reported previously^21^. Fecal excretion of MAP in feces was confirmed using the mycobacterial culture method by the Laboratoire d’épidémiosurveillance animale du Québec (Saint-Hyacinthe, Québec, Canada) as described (Fock-Chow-Tho et al., 2017). The cows were classified based on MAP excretion level and values obtained from both fecal PCR using the commercially USDA licensed VetMAX-Gold MAP Detection kit (where a Cq value for MAP DNA < 37 is considered positive) and serum ELISA using the IDEXX MAP Ab test kit (where S/P value >55 is considered positive), indicate a MAP-positive sample. The cows were then purchased from the dairy owners and housed in our barn (biosafety of level 2) for 1–3 weeks before euthanasia. Four cows positive to both serum ELISA and fecal culture tests (MAP+/+), five cows with positive serum ELISA but negative fecal culture test (MAP +/-), and five healthy cows, negative for both serum ELISA and fecal culture tests (MAP-/-), were categorized as JD positive (JDP), MAP tolerant (MAPT), and healthy control (HC) groups, respectively. Animals were humanely euthanized by intra-venous administration of 5 mg detomidine and 120 mL euthansol. The cows ranged in age from 3 to 7 years at the time of euthanasia. Intestinal tissues including ileum (IL), ileum or ileal lymph node (ILLN) and liver were collected, washed with phosphate buffered saline, cut into small pieces and immediately snap-frozen in liquid nitrogen before storage at -80 °C.

### RNA isolation

To isolate RNA, tissue sample pieces were removed from liquid nitrogen, weighed and about 30 mg tissue per sample was homogenized in 700 µL TRIzol Reagent (Life Technologies) using a Polytron homogenizer (Polytron PT 10–35 GT, Kinematica AG, Luzern, Switzerland) with a 7 mm probe for 10 s at 12,000 rpm. Total RNA was extracted using miRNeasy Kit (Qiagen Inc., Toronto, ON, Canada) according to the manufacturer’s protocol. Total RNA was subjected to DNase treatment using Turbo DNA-free™ Kit (Ambion Inc. Foster City, CA, USA). The concentration and integrity of total RNA were measured by Nanodrop ND-1000 (NanoDrop Technologies, Wilmington, DE, USA) and Agilent 2100 Bioanalyzer (Agilent Technologies, Santa Clara, CA, USA) using RNA 6000 Nano Labchip Kit (Agilent Technologies), respectively. RNA samples with an RNA integrity number (RIN) ≥ 7.0 were used for miRNA-Seq library preparation.

### Library construction and sequencing

The preparation of miRNA-Seq libraries was done according to Do et al.^69^. Following adapter ligation and amplification of the libraries, separation on polyacrylamide gel electrophoresis was done to size select the desired libraries with inserted miRNA. After elution from the gel, the libraries were concentrated by DNA clean and concentrator-5 kit (Zymo Research, Irvine, CA, USA). The quality and quantity of purified libraries were measured by the Picogreen assay (Life Technologies, Waltham, MA, USA) and a Nanodrop 3300 fluorescent spectrophotometer (NanoDrop Technologies), and then qPCR was used to further validate the concentration of each library for Illumina platforms (KAPA Biosystems, Wilmington, MA, USA). Libraries were multiplexed and sequenced according to Illumina’s suggested protocol by the Centre for Applied Genomics and the Hospital for Sick Children (Toronto, Canada, http://www.tcag.ca/) on an Illumina HiSeq 2500 platform. Sequencing on an Illumina HiSeq 2500 platform was performed as single-end 50-bases reads.

### MiRNA-Seq data analysis

The standard pipeline for small RNA sequencing data (smrnaseq, v2.2.0) from nf-core (https://nf-co.re/smrnaseq) was used to process the raw sequencing data. In brief, the quality control of raw sequence data was performed with FastQC program v0.12.0 (https://www.bioinformatics.babraham.ac.uk/projects/fastqc/). Adaptor sequences were trimmed with Trim Galore! V0.6.5 (https://www.bioinformatics.babraham.ac.uk/projects/trim_galore/), and reads were filtered using Bowtie2 v2.5.0 (https://bowtie-bio.sourceforge.net/bowtie2/index.shtml). Clean reads were collapsed and aligned to miRBase mature miRNA (v22.1) and the bovine reference genome (ARS-UCD1.2) using bowtie 1.3.0 (https://bowtie-bio.sourceforge.net/index.shtml).

### Identification of known MiRNA and novel MiRNA discovery

MiRBase v22.1 (http://www.mirbase.org/) was used to identify known miRNAs^70^. The quantifier.pl module from miRDeep2 (v2.0.1.2) (https://github.com/rajewsky-lab/mirdeep2) was used to discover novel miRNAs^71^. Novel miRNAs with ≥ 2 MiRDeep2 score and a significant randfold *p* value from miRDeep2 as well as with at least ten total read counts in each group were considered truly expressed in corresponding tissues and retained for further analyses.

### Differential expression analysis

Differential miRNA expression was analyzed with DESeq2 (v1.42.0) software (https://bioconductor.org/packages/release/bioc/html/DESeq2.html). The normalized counts of miRNAs were compared across three group comparisons, including JDP vs. HC, MAPT vs. HC and JDP vs. MAPT. Due to high inter cow variation and low sample size, a non-corrected *p*-value < 0.05 and |log2 fold change (log2FC)| >1 were used to define the significant differentially expressed (DE) miRNAs between groups.

### Prediction and enrichment of MiRNA target genes

The target genes of DE miRNAs per tissue were predicted with TargetScanHuman8.0 (https://www.targetscan.org/vert_80/). The target genes of miRNAs not included in the database of TargetScanHuman7.2 were predicted by using Perl scripts (targetscan_70.pl and targetscan7_context_scores.pl) (https://www.targetscan.org/cgi-bin/targetscan/data_download.vert80.cgi ). The cumulative weighted context + + score < -0.2 and context + + scores above 95th percentile were set as the threshold to filter the predicted target genes. Only target genes that passed the filter and were also expressed in the same tissues^45^ were further subjected to correlation analysis between DE miRNAs and predicted target genes by using Spearman’s rank correlation coefficient. Target genes with moderate and greater correlation (|rho| >0.3 and Benjamini-Hochberg procedure (FDR, false discovery rate) < 0.05) with their corresponding DE miRNAs were then used for gene ontology (GO) and KEGG pathways enrichment^72–74^ with ClueGO v2.5.10 (http://apps.cytoscape.org/apps/cluego)^75,76^. Significantly enriched GO terms and KEGG pathways were defined as having FDR < 0.05. The clustering of GO terms and KEGG pathways into functional groups was measured by kappa score that shows how they are similar with their associated genes. The overlap between enriched GO terms and KEGG pathways were visualized with the R packages “ggplot2” (v3.5.2) and “UpSet” (v1.4.0).

### Validation of MiRNA expression by real time quantitative PCR (qPCR)

Three DE miRNAs and one non-DE miRNA were randomly selected for the validation of their expression levels in IL. The same total RNA used for miRNA sequencing was reverse transcribed with the Universal cDNA Synthesis Kit II from Qiagen (Qiagen). The quantitative qPCR was performed with ExiLENT SYBR^®^ Green Master Mix Kit (Qiagen, ) and miRNA-specific miRCURY LNA™ Assays for each miRNA (Qiagen, ) according to the manufacturer’s instructions. Real time qPCR was performed on a StepOne Plus System (Applied Biosystems, Foster City, CA, USA). UniSp6 was used as internal control and the relative expression of miRNAs was calculated using the comparative Ct (2^−∆∆Ct^) method^77^.

## Result

### MiRNAs expressed in response to MAP infection in the ileum, ileum lymph node and liver tissues

The MAP infection status detection among enrolled commercial farms revealed four cows positive for MAP, five cows tolerant to MAP, and five healthy cows without MAP infection, which were categorized as JD positive (JDP), MAP tolerant (MAPT), and healthy control (HC) groups, respectively. Following the tissue collection, IL and liver tissue samples from one cow in the JDP group and liver tissues from four cows (two from MAPT and two from the JDP) were not available (Table S1-A). Finally, a total of 37 tissues from 14 Canadian Holstein cows, including 13 IL, 14 ILLN and 10 liver tissue samples, were used for small RNA sequencing, which generated about 12.5 million reads per tissue sample with an average GC content of about 55.49% (Table S1-B).

A total of 441 miRNAs were identified in IL, comprising 312 known miRNAs and 129 novel miRNAs (Table S2-A, D), while 405 (302 known and 103 novel) miRNAs were identified in ILLN (Table S2-B, D). In the liver, a comparatively lower number of miRNAs were identified, totaling 180 known and 21 novel miRNAs (Table S2-C, D). Notably, 28 miRNAs in IL, 30 miRNAs in ILLN and 14 miRNAs in liver showed high expression levels, surpassing 10,000 read counts per sample on average in the respective tissues. Of significance, 12 miRNAs were found to be highly expressed across all three tissues (Table 1). In IL, the top three miRNAs with the highest mean expression levels in all samples were bta-miR143, bta-miR145, and bta-miR192, each averaging more than 100,000 read counts per sample. Bta-miR-143 was the most highly expressed miRNA in both IL and ILLN, and the second most highly expressed miRNA in liver, bta-miR-122 being the highest expressed miRNA in the liver with mean read counts of 247,570 (Table S2C). The expression level of bta-miR-143 was extremely high in IL, reaching approximately 1.5 million read count per sample. In contrast, its expression was lower in ILLN and liver, with average read counts of 542,314 and 179,080 respectively. Similarly, bta-miR-145 ranked as the second most highly expressed miRNA in IL, with an average read count of 312,283. In ILLN, bta-miR-145 ranked third, and in the liver, it ranked fourth. Other highly expressed miRNAs also showed highest expression level in IL compared to ILLN and liver, such as bta-miR-192, bta-miR-100, bta-miR-30e-5p, bta-miR-125b (Table S2). Besides, bta-miR-2285f, the second most highly expressed miRNA, along with bta-miR-127 and bta-miR-99a-5p showed highest expression level in ILLN compared to IL and liver (Table 1).

**Table 1:** The average read counts of MiRNAs with high expression levels (> 8000 read counts) in all three tissues.

miRNA	IL				ILLN				Liver			
	All	HC	MAPT	JDP	All	HC	MAPT	JDP	All	HC	MAPT	JDP
bta-miR-143	1,549,209	2,046,066	1,297,648	1,140,384	542,314	556,763	587,143	468,216	179,080	235,174	110,647	141,494
bta-miR-145	312,283	442,685	230,695	230,927	79,718	94,224	73,113	69,843	36,388	48,327	21,228	29,280
bta-miR-192	157,957	197,605	138,684	123,999	13,829	6425	13,736	23,201	29,367	31,494	25,534	29,799
bta-miR-100	62,900	89,846	52,269	35,710	23,473	28,888	26,570	12,835	32,030	34,470	29,423	29,839
bta-miR-127	49,546	65,042	46,851	28,211	50,570	61,739	58,467	26,736	31,604	38,890	22,126	27,607
bta-miR-3600	45,762	63,537	35,307	33,564	37,522	38,585	32,002	43,093	52,717	56,755	56,284	37,272
bta-miR-30e-5p	35,568	38,730	33,137	34,348	33,884	24,816	42,240	34,772	17,063	17,397	17,110	16,156
bta-miR-2285f	31,469	27,133	38,899	26,310	81,490	109,788	77,550	51,044	10,821	11,416	10,862	9268
bta-miR-30a-5p	27,887	42,503	21,448	14,260	19,420	15,727	23,988	18,326	36,185	39,609	32,969	32,451
bta-miR-99a-5p	27,881	37,961	26,492	13,396	52,025	53,345	74,665	22,075	16,257	15,987	16,808	16,103
bta-miR-125b	20,885	31,477	14,214	14,352	18,378	22,819	20,122	10,646	10,338	11,845	8186	9800
bta-miR-22-3p	11,059	13,793	9634	8880	10,446	9018	10,858	11,717	12,611	14,052	12,981	8453

Note: The miRNA expression levels shown in this table is the average normalized read count in the corresponding groups. IL: ileum; ILLN: ileal lymph node; All: average for all treatments per group; HC: healthy control; MAPT: *Mycobacterium avium* subspecies paratuberculosis tolerant; JDP: Johne’s disease positive.

The highly expressed miRNAs with more than 10,000 read counts in IL were significantly enriched in 35 GO terms, including 30 biological processes (BP), 1 cellular component (CC), and 4 molecular function (MF) terms, organized into 17 groups (Table S3A, Figure S1A). The most significant groups were linked to the regulation of the extrinsic apoptotic signaling pathway and vascular permeability, both crucial for maintaining tissue integrity. In the ILLN, highly expressed miRNAs with more than 10,000 read counts were enriched in 136 BP, 4 CC, and 8 MF terms, clustered into 27 groups (Table S3B, Figure S1B). Six groups contained more than 10 GO terms, related to developmental processes, immune response, and hormone secretion. In contrast, only five GO terms were enriched for the highly expressed miRNAs with more than 10,000 read counts in the liver, with liver development (GO:0001889) being a key term (Table S3C).

### Differentially expressed MiRNAs in the IL, ILLN and liver during subclinical johne’s disease

**Fig. 1:**
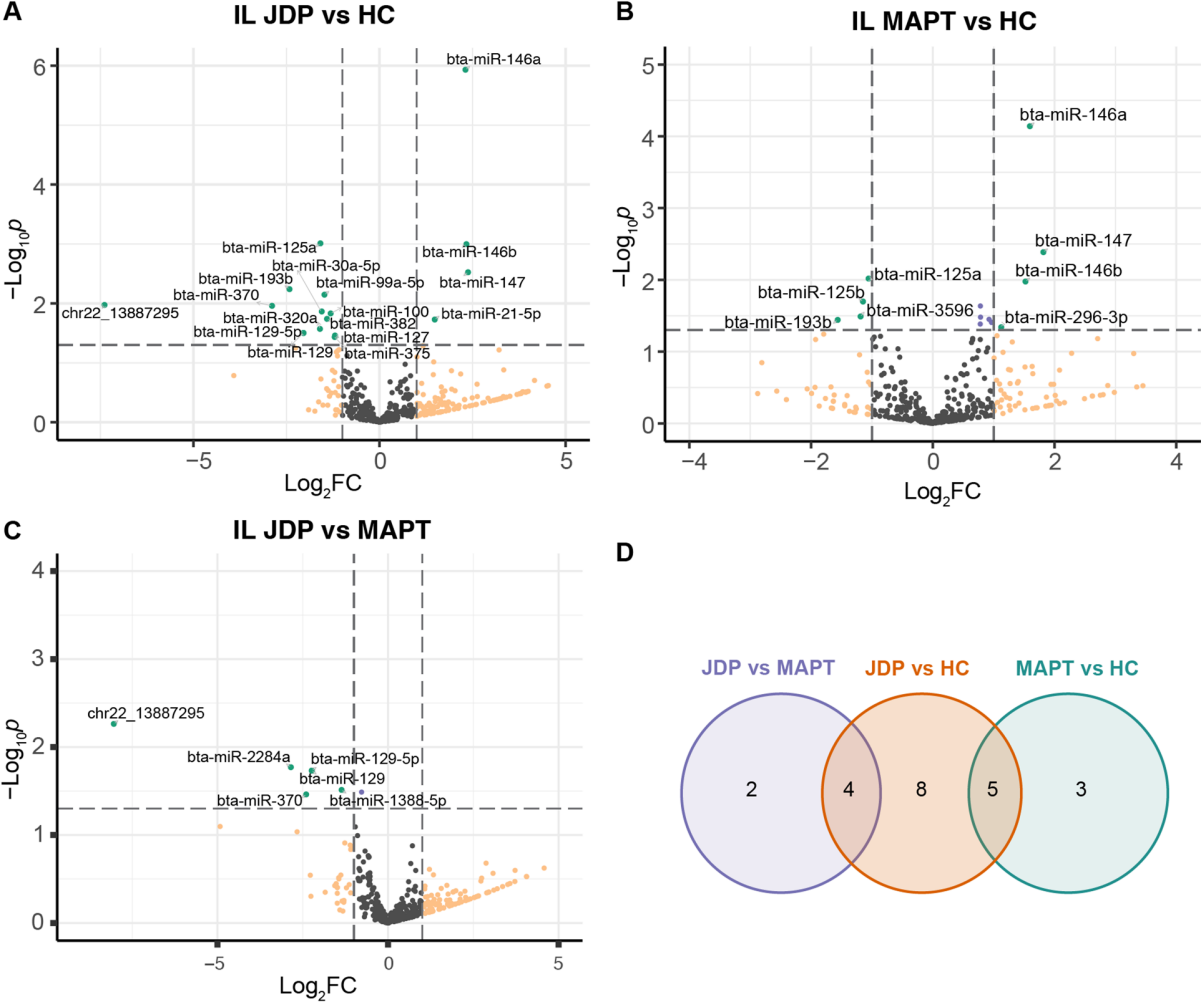
Differentially expressed miRNAs identified in IL tissue of cows with subclinical Johne’s disease. (**A-C**) Volcano plot showing the DE miRNAs identified in IL by comparing JDP vs. HC (**A**), MAPT vs. HC (**B**) and JDP vs. MAPT (**C**) groups, respectively. (**D**) Venn plot showing the intersect of DE miRNAs identified by the three comparisons. DE: differentially expressed; IL: ileum; JDP: Johne’s disease positive; MAPT: *Mycobacterium avium* subspecies paratuberculosis tolerant; HC: healthy control; FC: fold change.

A total of 22 miRNAs exhibited significant differential expression (DE) (*p* < 0.05, |log2FC > 1|) in IL tissue across three comparison groups during JD (Fig. 1, Table S4A). Comparison of JDP group to HC group revealed 17 DE miRNAs, including 4 up-regulate and 13 down-regulated miRNAs (Fig. 1A). Additionally, 8 DE miRNAs were identified between MAPT and HC (Fig. 1B, Table S4B), while 6 miRNAs were differently expressed between JDP and MAPT groups (Fig. 1C, Table S4C). Notably, five miRNAs (bta-miR-146a, bta-miR-125a, bta-miR-146b, bta-miR-147 and bta-miR-193b) having the same direction of expression (up- or downregulated) but with slightly higher levels in JDP group were differentially expressed between both JDP vs. HC and MAPT vs. HC groups (Fig. 1D). Remarkably, bta-miR-146a, bta-miR-146b, bta-miR-147, bta-miR-125a, and bta-miR-193b exhibited significantly higher expression levels in both JDP and MAPT groups compared to HC (Fig. 1D, Table S4H, summary), but their expression levels were not different for the JDP vs. MAPT comparison group. Furthermore, novelmiR_22_13887295, bta-miR-370, bta-miR-129 and bta-miR-129-5p were identified as DE miRNAs in both the comparisons of JDP vs. HC and JDP vs. MAPT (Fig. 1D, all showing significantly down-regulated expression level. Notably, the miRNA novelmiR_22_13887295 emerged as the sole novel DE miRNA displaying the most substantial changes with a log2FC of -7.38 and − 8.04 in the JDP compared to HC or MAPT groups, respectively.

**Fig. 2:**
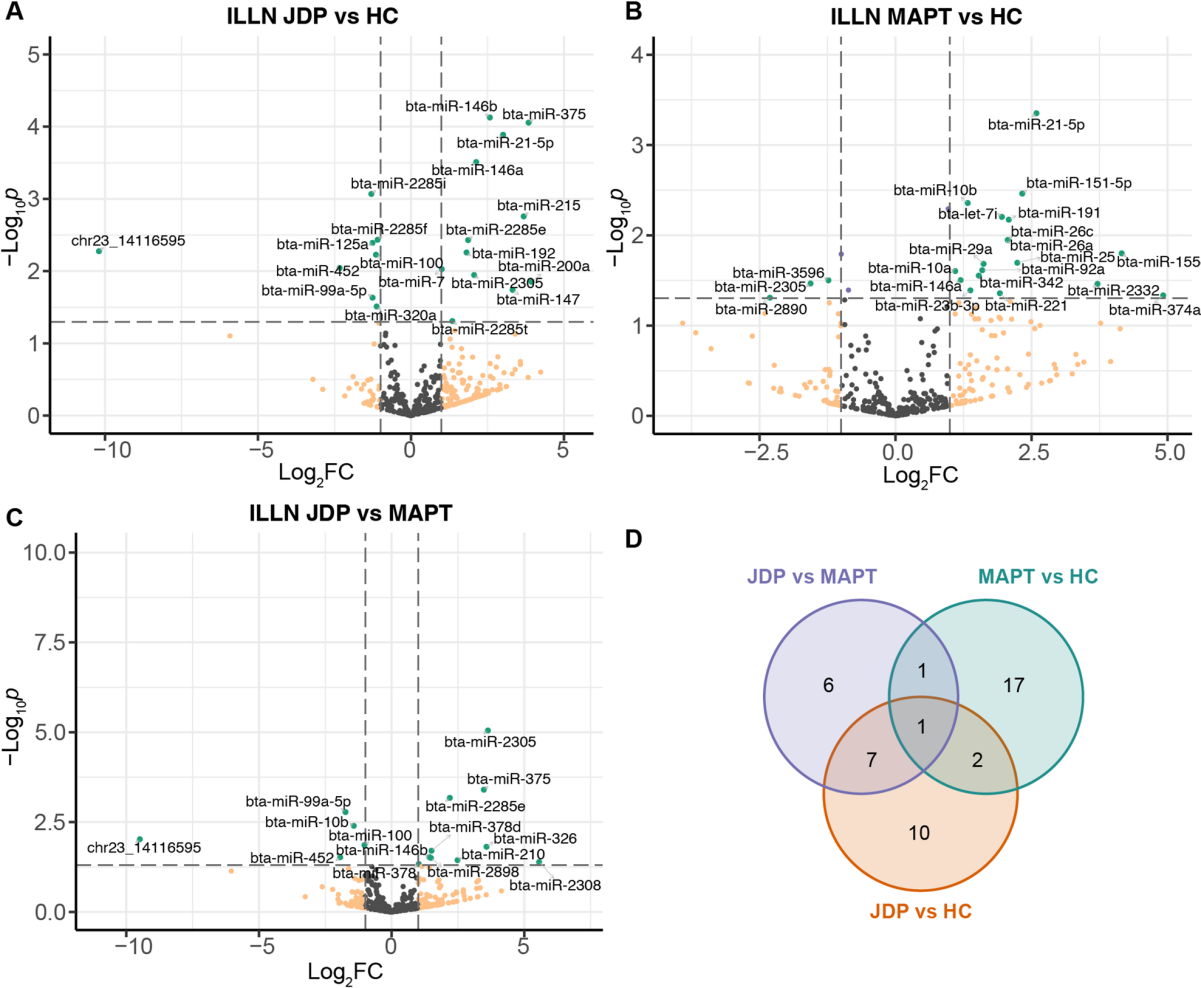
Differentially expressed miRNAs identified in ILLN of cows with subclinical Johne’s disease. (**A-C**) Volcano plot showing the DE miRNAs identified in ILLN by the comparison JDP vs. HC (**A**), MAPT vs. HC (**B**) and JDP vs. MAPT (**C**), respectively. (**D**) Venn plot showing the intersect of DE miRNAs identified by three comparisons. DE: differentially expressed; ILLN: ileal lymph node; JDP: Johne’s disease positive; MAPT: *Mycobacterium avium* subspecies paratuberculosis tolerant; HC: healthy control; FC: fold change.

A higher number of miRNAs (*N* = 44) exhibited significant differential expression in ILLN in response to MAP infection status. Specifically, 20 (12 up-regulated and 8 down-regulated), 21 (18 up-regulated and 3 down-regulated), and 15 DE miRNAs (10 up-regulated and 5 down-regulated), were identified in the comparison of JDP vs. HC (Fig. 2-A), MAPT vs. HC (Fig. 2B) and JDP vs. MAPT (Fig. 2C), respectively (Table S4D-F). Remarkably, bta-miR-2305 was identified as DE miRNA in all three comparisons (Fig. 2D, Table S4H), demonstrating significant expression differences among the three groups, with the highest and lowest expression levels in JDP and MAPT groups respectively. In addition to bta-miR-2305, another seven miRNAs were identified in both JDP vs. HC and MAPT vs. HC comparisons, including bta-miR-99a-5p, bta-miR-100, bta-miR-146b, bta-miR-375, bta-miR-452, bta-miR-2285e, and novelmiR_23_14116595 (Table S4H). Out of the 15 miRNAs DE for the JDP vs. MAPT comparison in the ILLN, 8 (bta-miR-100, bta-miR-146b, bta-miR-2285e, bta-miR-375, bta-miR-452, bta-miR-99a-5p, novelmiR_23_14116595 and bta-miR-2305) were also found as DE in the JDP vs. HC comparison and 2 (bta-miR-2305 and bta-miR-10b) in the MAPT vs. HC comparison (Fig. 2D). Furthermore, bta-miR-10b identified as DE miRNA in the comparison of MAPT vs. HC and MAPT vs. JDP, exhibited lower expression in the JDP group compared to MAPT (log2FC = -1.43) but higher expression in the MAPT group than the HC group (log2FC = 1.33). Bta-miR-146a and bta-miR-21-5p were both significantly up-regulated in JDP and MAPT groups compared to HC group.

Interestingly, 11 DE miRNA were identified in both IL and ILLN (Table 2). Specifically, bta-miR-146b, bta-miR-21-5p, bta-miR-146a and bta-miR-147 were up-regulated, while bta-miR-375, bta-miR-125a, bta-miR-100, bta-miR-99a-5p and bta-miR-320a were down-regulated in JDP group compared to HC group in both IL and ILLN. Similarly, bta-miR-146a was identified as up-regulated, while bta-let-7b also known as bta-miR-3596 was identified as down-regulated in MAPT group compared to HC group in both IL and ILLN.

**Table 2:** Differentially expressed MiRNAs identified in JDP or MAPT cows compared to healthy cows.

Comparison	miRNA	IL		ILLN	
		Log2FC	*p* value	Log2FC	*p* value
JDP vs. HC	bta-miR-146b	2.334	0.001	2.580	0.000
	bta-miR-375	-1.205	0.037	3.845	0.000
	bta-miR-21-5p	1.480	0.020	3.012	0.000
	bta-miR-146a	2.310	0.000	2.133	0.000
	bta-miR-125a	-1.588	0.001	-1.260	0.000
	bta-miR-100	-1.313	0.015	-1.145	0.006
	bta-miR-147	2.380	0.000	3.323	0.018
	bta-miR-99a-5p	-1.479	0.007	-1.255	0.023
	bta-miR-320a	-1.600	0.027	-1.121	0.031
MAPT vs. HC	bta-miR-146a	1.523	0.011	1.200	0.030
	bta-let-7b	-1.187	0.032	-1.240	0.030
	bta-miR-3596	-1.190	0.030	-1.240	0.030

The three differentially expressed miRNAs (bta-miR-2483-3p, bta-miR-103 and bta-miR-2305) identified in liver were not identified in IL or ILLN tissues. IL: ileum, ILLN: ileum lymph node, JDP: Johne’s disease positive, HC: healthy cows, MAPT: *Mycobacterium avium* subspecies paratuberculosis tolerant.

In the liver, three miRNAs were DE for the comparisons JDP vs. HC and/or JDP vs. MAPT. As liver samples were only collected from two cows in the JDP group, this result will be referred to minimally in this study (Table S4G).

### Predicted MiRNA target genes

Firstly, the target genes of the DE miRNAs were predicted using TargetScan and filtered against mRNAs expressed in the same samples. The mRNA data has been reported previously^45^. Then, correlation analysis was performed between the expression levels of the mRNAs predicted by TargetScan and expressed in the same samples and the expression levels of DE miRNAs. Only target mRNAs displaying at least a moderate correlation with the corresponding DE miRNAs were retained as the predicted target genes of the DE miRNAs (Table S5). In the IL, a total of 279, 210 and 71 target genes were predicted for DE miRNAs identified in JDP vs. HC, MAPT vs. HC and JDP vs. MAPT comparisons, respectively (Table S5A-C). Importantly, a noteworthy observation was the strong negative correlation observed between DE miRNAs and some of their predicted target genes, providing further evidence for the potential impact of miRNA on their expression. For instance, the down-regulated bta-miR-125a and bta-193b in both JDP and MAPT groups when compared to HC group, exhibited a robust negative correlation with 11 genes (*AGTRAP*, *ATOX1*, *DAZAP2*, *GAA*, *GGA2*, *GNA15*, *GPATCH3*, *IL7R*, *ITGB2*, *PPT1*, and *TNFSF8*) and 4 genes (*KIAA1919*, *MAFG*, *PTP4A2*, and *ZBTB6*), that displayed higher expression level in JDP or MAPT group than HC group (*r* < -0.7), respectively (Table S5A-B). Besides, the down-regulated novel DE miRNA novelmiR_22_13887295 (exhibited significantly lower expression in the JDP group compared to both the MAPT and HC groups) displayed a notable and significant negative correlation with *ARL8B* (*r* < -0.7). In addition, significant negative correlations were also identified between bta-miR-21-5p, bta-miR-30a-5p, and bta-miR-320a and one (*ZNF772*), three (*DDIT4*, *SLC7A10*, and *SNX8*) and two (*TFRC* and *USP12*) of their predicted target genes, respectively, in the comparison JDP vs. HC (Table S5A), respectively. Conversely, the down-regulated bta-miR-125b in MAPT group, when compared to the HC group, demonstrated a significant negative correlation with *MAPK12* (i.e. up-regulation of gene expression).

In ILLN, 255, 179 and 280 target genes were predicted for the 20, 21 and 15 DE miRNAs identified in the comparisons of JDP vs. HC, MAPT vs. HC and JDP vs. MAPT, respectively (Table S5D-F). Bta-miR-2305, identified as a DE miRNA in all three comparisons with highest expression level in JDP group, exhibited a strong negative correlation with 19 target genes in ILLN (Table S5D-F). Bta-miR-2305 was also potentially up-reglued in JDP compared to MAPT in liver and significantly negatively correlated with *LYSMD3* and *SFRP2* (Table S5G). In addition, the down-regulated bta-miR-452 in JDP when compared to MAPT or HC, displayed a negative correlation with six genes (*CCR5*, *GNG7*, *SCP2*, *SCPEP1*, *SGPP1*, and *TGFA*), all of which had higher expression level in JDP group (Table S5D and F). Bta-miR-21-5p and bta-miR-2285e, which were up-regulated in both JDP and MAPT groups compared to HC, showed strong negative correlations with *NTF3* and *FLRT3*, respectively. Similarly, strong negative correlations were observed between DE miRNAs, including bta-miR-192, bta-miR-200a, bta-miR-2285t bta-miR-7, bta-let-7i, bta-miR-2898, bta-miR-326, bta-miR-378 and bta-miR-378d, and some of their predicted target genes in ILLN. These strong negative correlations strongly suggested their potential effects on the expression of their target genes in response to JD.

### Functional enrichment for DE MiRNAs

**Fig. 3:**
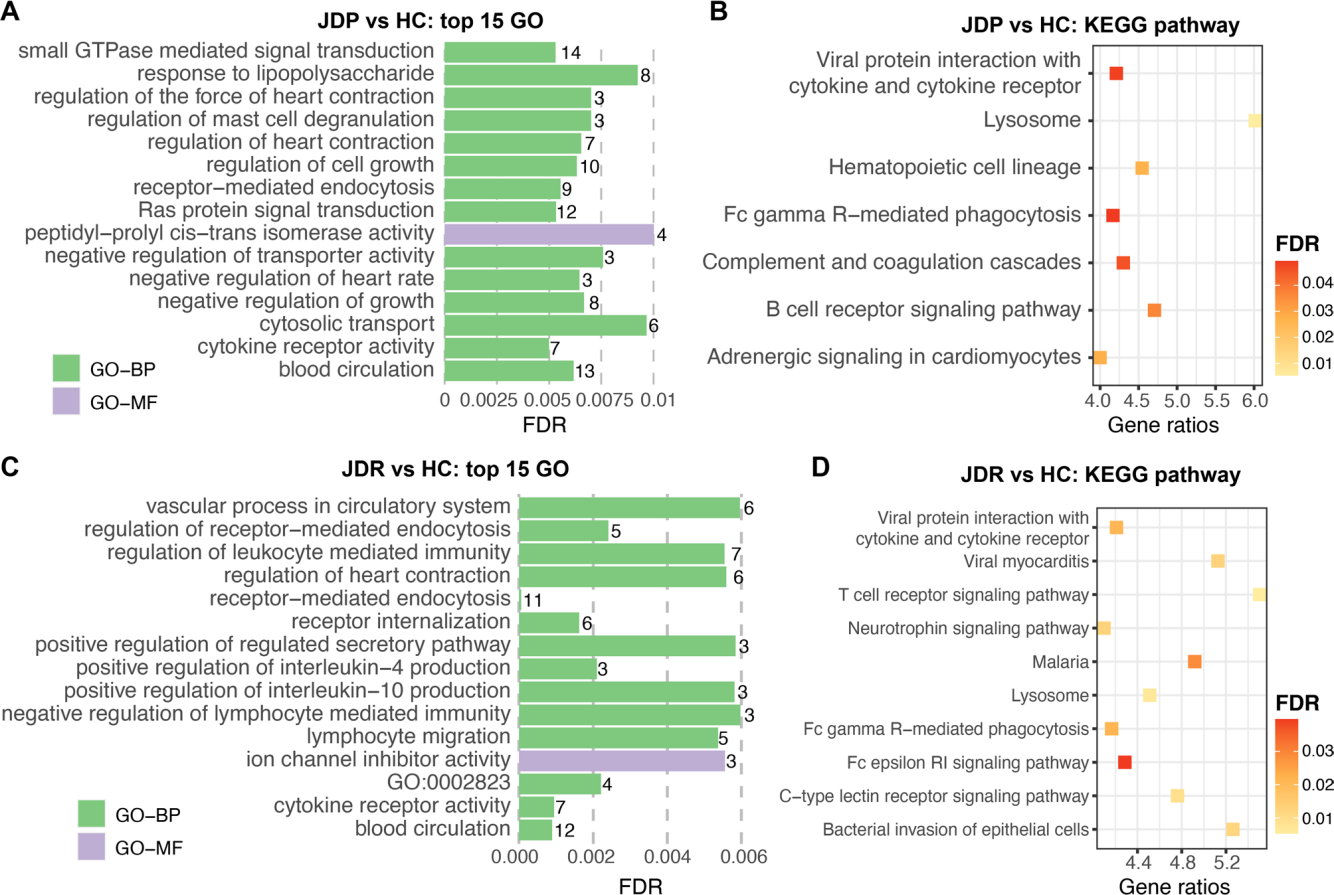
Functional enrichment for DE miRNAs identified in IL. (**A**) Top 15 most significant GO terms and (**B**) seven KEGG pathways significantly enriched by target genes of DE miRNAs identified in JDP compared to HC group; (**C**) Top 15 most significant GO terms and (**D**) ten significant KEGG pathways enriched by target genes of DE miRNAs identified in MAPT vs. HC groups. The number at the right side of each bar in (**A**) and (**C**) represents the number of target genes involved in the corresponding GO term. DE: differentially expressed; IL: ileum; JDP: Johne’s disease positive; MAPT: *Mycobacterium avium* subspecies paratuberculosis tolerant; HC: healthy control; FDR: false discovery rate; GO-BP: GO terms in the category of Biological Process; BO-MF: GO terms in the category of Molecular Function.

The 279 target gens of DE miRNAs identified in IL between JDP and HC groups were significantly enriched in 68 GO terms, including 60 BP-, 2 CC- and 6 MF- GO terms, as well as 7 KEGG pathways (Table S6A). The most significantly enriched GO term and KEGG pathway with smallest FDR were cytokine receptor activity (GO:0004896, FDR = 0.005) and Lysosome (KEGG:04142, FDR = 0.005), respectively (Fig. 3A-B). These functional annotations, comprising both GO terms and KEGG pathways, were further categorized into 25 functional groups, with four groups featuring no fewer than five functional annotations (Table S6A). The largest group is related to blood circulation and transport activities, encompassing 10 BP-GO terms, 2 MF-GO terms and 1 KEGG pathway (“Group24”, Table S6A). The second-largest group, comprising 12 BP-GO terms (“Group23”, Table S5A), and the fourth group, “Group21” (4 BP-GO terms and 1 KEGG pathway, Table S6A) are processes related to leukocyte-mediated immunity and other immune- processes. While “Group22” (Table S6A) composed of nine BO-terms is related to cell growth processes.

The 210 target genes of DE miRNAs in IL identified between MAPT and HC groups were significantly enriched in 24 BP-GO terms, 3 MF-GO terms and 10 KEGG pathways, which were significantly clustered into 16 functional groups (Table S6B). The top three most significant GO terms are receptor-mediated endocytosis (GO:0006898, FDR = 5.96 × 10^− 5^), blood circulation (GO:0008015, FDR = 8.94 × 10^− 4^) and cytokine receptor activity (GO:0004896, FDR = 9.47 × 10^− 4^), respectively (Fig. 3C). T cell receptor signaling pathway (KEGG:04660, FDR = 0.0052) was the most significantly enriched KEGG pathway (Fig. 3D), which was one of eight functional annotations in the biggest functional group (“Group15”, Table S6B). The second biggest group, which is also the most significant group (FDR = 4.11 × 10^− 5^) included five BP-GO terms with endocytosis and thermogenesis related functions (“Group14”, Table S6B). Another group also composed of five BP-GO terms has functions related to the circulatory system (“Group13”, Table S6B). The other functional groups are smaller with less than five functional annotations (Table S6B). Besides, the 71 target genes of DE miRNAs of JDP vs. MAPT in IL were only significantly enriched in one BP-GO term, neural crest cell development (GO:0014032, FDR = 0.0015).

**Fig. 4:**
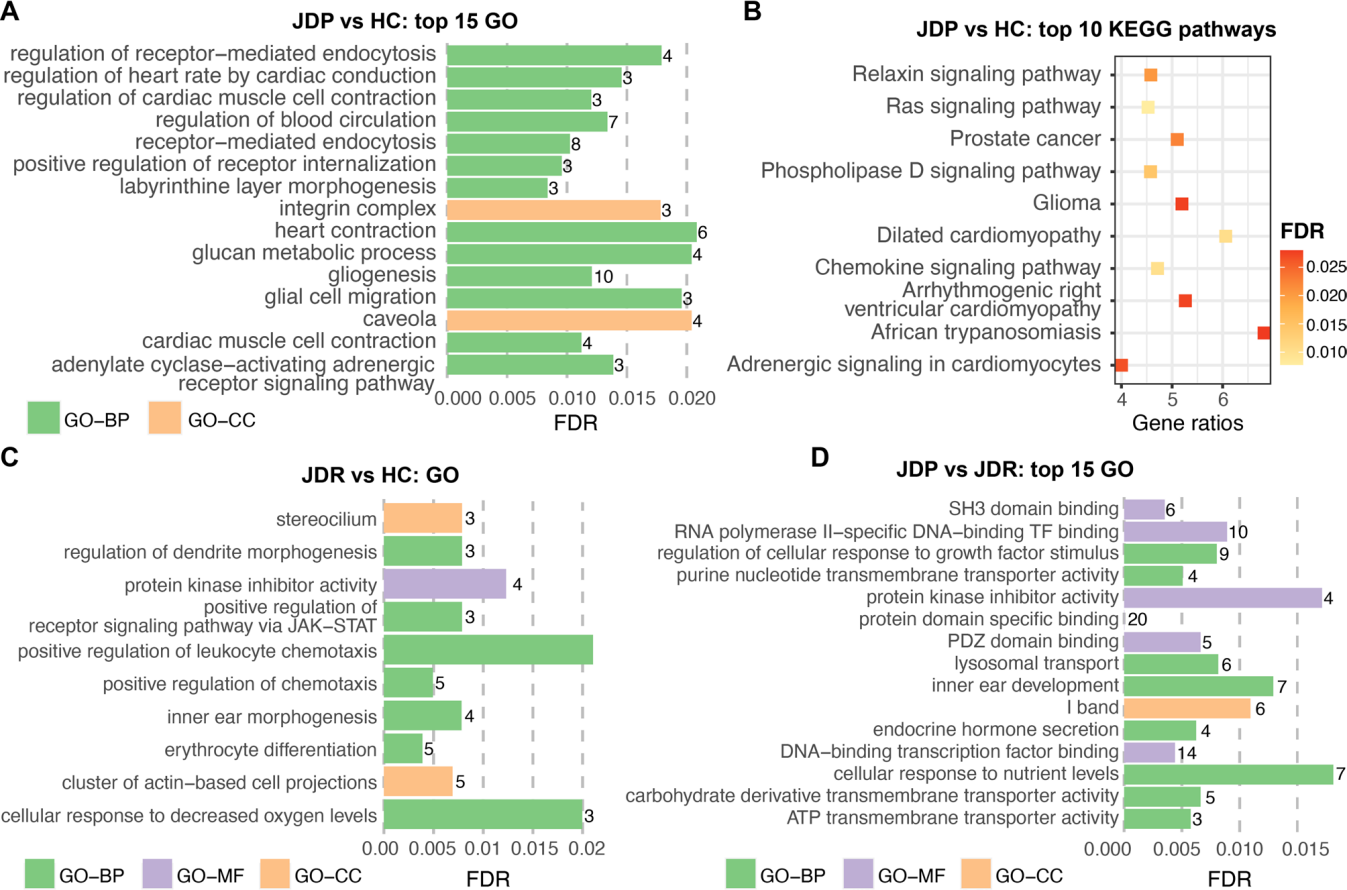
Functional enrichment for DE miRNAs identified in ILLN. (**A**) Top 15 GO terms and (**B**) top 10 KEGG pathways most significantly enriched by target genes of DE miRNAs identified in ILLN between JDP and HC groups; (**C**) Ten GO terms significantly enriched by target genes of DE miRNAs identified in ILLN between MAPT and HC groups. (**D**) Top 15 GO terms most significantly enriched by target genes of DE miRNAs identified in ILLN between JDP and MAPT groups. The number at the right side of each bar in (**A**), (**C**) and (**D**) represents the number of target genes involved in the corresponding GO term. DE: differentially expressed; ILLN: ileal lymph node; JDP: Johne’s disease positive; MAPT: *Mycobacterium avium* subspecies paratuberculosis tolerant; HC: healthy control; FDR: false discovery rate; GO-BP: GO terms in the category of Biological Process; BO-MF: GO terms in the category of Molecular Function; BO-CC: GO terms in the category of Cellular Component.

In the ILLN, a total of 27 GO terms (23 BP-, 2 MF- and 2 CC-GO terms) and 20 KEGG pathways were significantly enriched by the 255 target genes of DE miRNAs identified between JDP vs. HC groups (Table S6D). Labyrinthine layer morphogenesis (GO:0060713, FDR = 0.0084), positive regulation of receptor internalization (GO:0002092. FDR = 0.0096), and receptor-mediated endocytosis (GO:0006898, FDR = 0.0102) were the top three most significantly enriched GO terms. (Fig. 4A). The most significantly enriched KEGG pathway is Ras signaling pathway (KEGG:04014, FDR = 0.0077) followed by Chemokine signaling pathway (KEGG:04062, FDR = 0.0010) (Fig. 4B). The 47 functional annotations were clustered into 14 functional groups, including four groups with at least five GO terms and/or KEGG pathways (Table S6D). The biggest and most significant functional group included 7 BP-GO terms, 1 CC-GO term and 5 KEGG pathways, involved in heart contraction related processes (“Group13”, Table S6D). The next group is composed of 12 KEGG pathways, including the most significantly enriched Ras signaling pathway and Chemokine signaling pathway (“Group12”, Table S5D). “Group11” (Table S6D) merged as the third-biggest group composed of 3 BP-GO terms (regulation of response to wounding (GO:1903034), positive regulation of interleukin-8 production (GO:0032757) and positive regulation of type II interferon production (GO:0032729)) and 4 KEGG pathways (including African trypanosomiasis (KEGG:05143), Toxoplasmosis (KEGG:05145), Hypertrophic cardiomyopathy (KEGG:05410), and Viral protein interaction with cytokine and cytokine receptor (KEGG:04061)), involved in immune-related functions or diseases. “Group10” (Table S6D) with 5 BP-GO terms is involved in endocytosis-related processes and regulation.

The 171 target genes of DE miRNAs identified in ILLN MAPT vs. HC comparison were significantly enriched in less functional annotations, including 10 GO terms (Fig. 4C) and 2 KEGG pathways (FoxO signaling pathway (KEGG:04068, FDR = 0.0072) and Notch signaling pathway (KEGG:04330, FDR = 0.0160)) (Table S6E). The top three most significantly enriched GO terms were erythrocyte differentiation (GO:0030218, FDR = 0.0039), positive regulation of chemotaxis (GO:0050921, FDR = 0.0049) and cluster of actin-based cell projections (GO:0098862, FDR = 0.0069), respectively (Fig. 4C, Table S6E).

The DE miRNAs with 280 target genes in ILLN between JDP and MAPT groups were significantly enriched in 44 GO terms, including 22 BP-, 9 MF- and 13 CC-GO terms, and 5 KEGG pathways (Table S6F). The top 15 most significantly enriched GO terms are shown in Fig. 4D, with protein domain specific binding (GO:0019904, FDR = 2.20 × 10^− 5^) being the most significant. The 49 functional annotations were clustered into 25 functional groups, but with only two groups with five or more annotations (Table S6F).“Group24” (Table S6D) with the most annotations consisted of 6 CC-GO terms and 2 GO-terms, followed by “Group23” (Table S6D) with 3 CC-, 1 MF- and 1BP-GO term, are related to molecular and cellular components/processes, and have functions associated with muscle structure and function, particularly in the context of cardiac muscle cells.

**Fig. 5:**
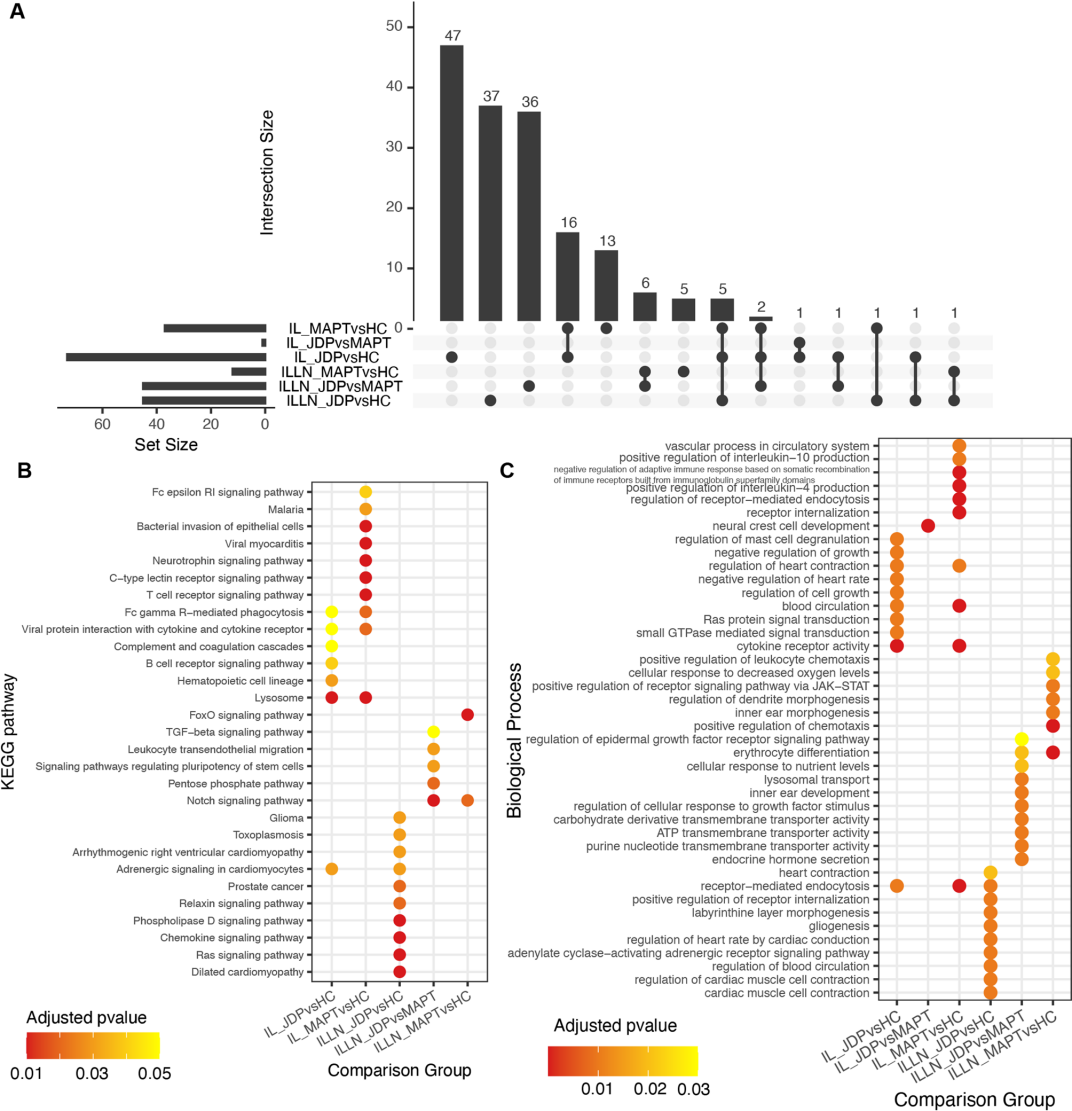
Comparison of enriched biological processes and pathways across experimental groups. (**A**) UpSet plot showing the intersection of enriched pathways across different comparison groups. Each set represents pathways enriched in a specific group, and the bars indicate the number of pathways unique or shared among groups. (**B**, **C**) Bubble plot illustrating the top 10 enriched KEGG pathways (**B**) and Biological Processes (**C**) per comparison group based on adjusted p-values. Dot color indicates the adjusted p value. Pathway terms are shown on the y-axis and comparison groups on the x-axis.

The majority of significant GO terms and KEGG pathways were uniquely enriched in each comparison, with only a small proportion shared between two or more comparisons (Fig. 5A). For example, five terms, including receptor-mediated endocytosis (GO:0006898), heart contraction (GO:0060047), receptor internalization (GO:0031623), glucan metabolic process (GO:0044042), and Viral protein interaction with cytokine and cytokine receptor (KEGG:04061), were enriched by DE miRNAs identified in three comparisons: JDP vs. HC and MAPT vs. HC in IL, and JDP vs. HC in ILLN. Additionally, regulation of heart contraction (GO:0008016) and ion channel regulator activity (GO:0099106) were enriched by DE miRNAs in the comparisons JDP vs. HC and MAPT vs. HC in IL, as well as JDP vs. MAPT in ILLN. Beyond these shared terms, DE miRNAs identified in the JDP vs. HC and MAPT vs. HC comparisons in IL were also commonly enriched in 14 additional GO-BP terms and 2 KEGG pathways, many of which are related to immune functions. These include cytokine receptor activity (GO:0004896), regulation of leukocyte-mediated immunity (GO:0002703), lymphocyte migration (GO:0072676), and negative 

 regulation of adaptive immune response based on somatic recombination of immune receptors built from immunoglobulin superfamily domains (GO:0002823) (Table S6). Furthermore, DE miRNAs from the MAPT vs. HC and JDP vs. MAPT comparisons in ILLN shared six enriched terms: erythrocyte differentiation (GO:0030218), cluster of actin-based cell projections (GO:0098862), regulation of dendrite morphogenesis (GO:0048814), positive regulation of receptor signaling pathway via JAK-STAT (GO:0046427), protein kinase inhibitor activity (GO:0004860), and the Notch signaling pathway (KEGG:04330). These accounted for half of all terms enriched in MAPT vs. HC in ILLN. However, only few of the top 10 enriched terms were common to two or more comparisons and tissues (Fig. 5B-C).

### Quantitative real time PCR (qPCR) validation of MiRNA expression

**Fig. 6:**
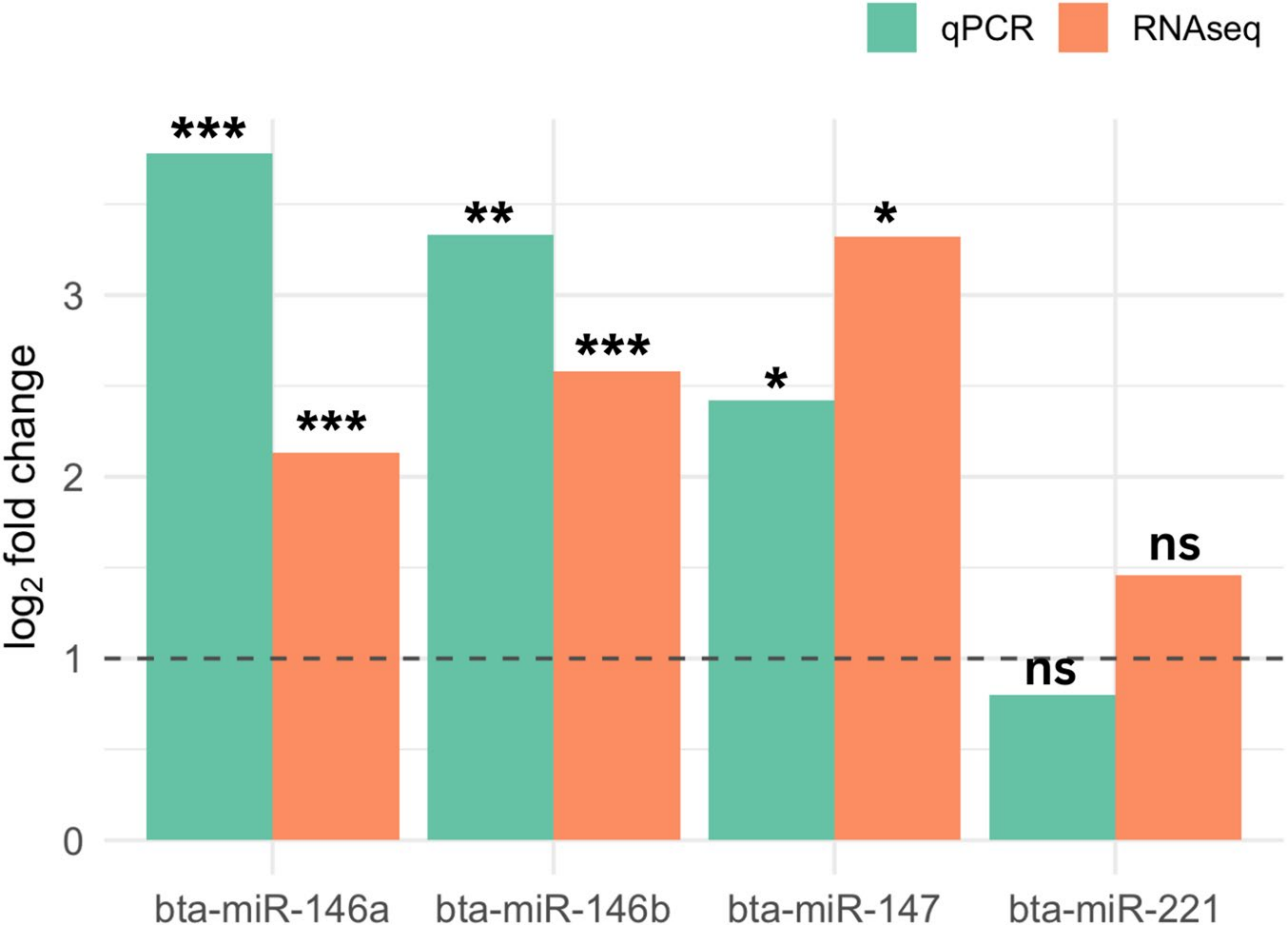
Relative expression of four miRNAs detected by RNA-seq and real time qPCR. Green and orange bar represent the relative expression changes of corresponding miRNAs between JDP and HC group detected by real time qPCR and RNA sequencing in ileal lymph node tissues, respectively. *=*p* < 0.05, **=*p* < 0.01, *** = *p* < 0.001, ns = not significant.

The real time qPCR detected higher expression levels of bta-miR-146a, bta-miR-146b, and bta-miR147 in JDP cows than HC cows in ILLN while similar expression levels of bta-miR-221 in two groups was observed (Fig. 6). This is consistent with the results detected by RNA-seq.

## Discussion

The main point of MAP entry is the small intestine where it may cause lesions in intestinal tissues and related lymphoid tissues, and progressively affects the absorption of nutrients leading to muscle wasting and lower productivity^78–80^. MiRNAs have the potential to regulate immune cell differentiation and host defense against pathogenic infections^81–83^ and have been suggested to have important regulatory roles in MAP infection^37,50,54,55,59,60,84^. Therefore, exploration of miRNA expression patterns in cow intestinal ileal tissue (IL) and related mesenteric lymphoid tissue (ILLN) from cows with JD is an avenue to uncover the potential regulatory roles of miRNAs in response to naturally occurring MAP infection in Holstein cows.

The miRNA profiles were different for the IL, ILLN and liver tissues, with more miRNAs expressed in the IL (441) and ILLN (405) than in the liver (201). Considering the miRNA expression in all samples per tissue, bta-miRNA-143 was the most abundantly expressed in the IL and ILLN and the second most expressed in the Liver, with significantly higher expression level in IL (base mean: 1,459,209) as compared to ILLN (542,314) and liver (179,080). This confirms previous studies that reported bta-miR-143 as the most abundantly expressed miRNA in several bovine tissues including ileum, rumen, skeletal muscle and testis^62,63,85,86^. These reports suggested important roles for miR-143 in development processes such as cell differentiation and tissue development, thus confirming a potential role in the regulation of developmental processes and other related processes in the cow intestine. The expression of miR-143 was also reported in most bovine tissues investigated^87–89^. Bta-miR-145, the second most abundantly expressed miRNA in IL and liver, and the third in ILLN was reported as downregulated in ileocecal valve with diffuse vs. focal lesions from MAP infected cows^54^ as well as to cooperate with bta-miR-143 to regulate the quiescent and proliferative phenotype of smooth muscle cell in the mouse^90,91^. Important roles for bta-miR-143 and bta-miR-145 in the host response to diseases have been revealed for ulcerative colitis^92^, colorectal cancer^93^ and breast cancer^94^. Bta-miR-192, the third most abundantly expressed miRNA in the IL, and also highly expressed in ILLN and liver, has important roles in immune response to *Escherichia coli* infection in cattle^95^ and associations with regulation of small cell lung cancer and colorectal cancer in humans^96,97^. Bta-miR-122, the most highly expressed in the liver, is a liver specific miRNA with main function of maintaining homeostasis of the liver, including regulation of hepatic cholesterol and fatty acid metabolism, and it dysregulation has been found to play critical roles in liver disease and infections caused by viral, bacterial and parasitic pathogens^98,99^.

This study identified miRNAs that were highly expressed in the different tissues, including 12 highly expressed miRNAs common to all tissues, demonstrating specific and common regulatory roles (Table 1 and Table S3). The functional enrichment of the 28 highly expressed miRNAs in the IL suggests roles in maintaining tissue homeostasis, regulating vascular processes like blood circulation and vascular permeability, and facilitating carbohydrate and fatty acid metabolism. Observed changes in lipid homeostasis and fatty acid and carbohydrate metabolism of macrophages from JD cows^41^ could be explained by the role of miRNAs in disease pathology. Primary macrophages infected with MAP have been described with compromised apoptosis^41^, which could be explained by the role of these miRNAs as they modulate the extrinsic apoptotic signaling pathway, contributing to cell survival and immune regulation, including the negative regulation of T cell-mediated immunity. These findings highlight miRNAs’ importance in balancing nutrient absorption, immune response, and epithelial integrity in the ileum. The highly expressed miRNAs in the ILLN (*n* = 30) play significant roles in immune response regulation, particularly in processes like macrophage activation, T cell differentiation, leukocyte migration, and type 2 immune response. These miRNAs also influence lipid metabolism and biosynthetic pathways, such as cholesterol biosynthesis and fatty acid biosynthesis, indicating their role in maintaining metabolic balance during immune responses. The 14 highly expressed miRNAs in the liver are associated with liver development, protein kinase inhibition, and protein phosphatase regulation, influencing cellular signaling. They also play a role in protein processing between the endoplasmic reticulum and Golgi, and help regulate immune responses by suppressing leukocyte differentiation.

The most DE miRNAs in this study were identified in ILLN (*n* = 45), followed by IL (24) and liver (*n* = 3). As shown in Figs. 1D and 2D, only few DE miRNAs were common between two or more comparisons. It has been noted that miRNAs demonstrate tissue specificity in different cell types associated with specific functions^100,101^. These results corroborate our earlier findings demonstrating tissue specific responses to MAP infection with more DNA methylation alterations (differentially methylated cytosines and differentially methylated regions) in the ILLN compared to IL^100^, suggesting epigenetic regulation of gene expression in the ILLN during MAP infection. Further support to this observation is the observation of a higher number of DE genes involved in the immune and diseases processes in the IL (heightened immune state) compared to ILLN (dampened immune state)^45^, suggesting that the higher DNA methylation alterations^102^ and higher DE miRNAs (this study) contributed to suppress the immune state of the ILLN or regulate/resolve disease processes in the ILLN compared to the IL.

In the IL, majority of the DE miRNAs observed for the JDP vs. HC comparison were downregulated (13 DE miRNAs out of 17) (Table S4A). Many of these miRNAs have been found to be involved in livestock bacterial and viral infections^52,103^. It is worthy of note that, bta-miR-100 was lowly expressed in IL (log_2_FC = -1.31) and ILLN (log_2_FC= -1.14) of JDP group compared to HC, and in ILLN (log2FC = -1.02) of JDP group compared to MAPT group. Down regulation of bta-miR-100 has been observed in the mammary tissue of cows in response to *Streptococcus uberis* infection in cattle^104^ and to interact with pathogen sensing during the first weeks of calf’s small intestinal development^40^. Reduced bta-miR-100 expression was also reported in whole blood of MAP infected Holstein cows and was selected as a possible biomarker of MAP infection^37^. These studies and our data suggest important roles for bta-miR-100 in the regulation of MAP infection. Bta-miR-370, another down-regulated miRNA in IL in JDP and MAPT groups compared to HC, (Log_2_FC = -2.89 and − 2.40, respectively) is correlated with genes that impacts lipid metabolism in primary macrophages from JD cows^41^ and it is implicated in diseases, including type 2 diabetes in mice^105^. Interestingly, bta-miR-370 might impact as well the expression of genes (*SERBP-1*, *DGAT2* and Cpt1α) that affect the accumulation of hepatic triglycerides and lipid metabolism in mice^106^ similar to the pathways affected in primary macrophages from JD cows^41^. MiR-370 was also found to inhibit cell growth and metastasis in tumor cells^107^.

Interestingly, bta-miR-146a, bta-miR-146b, and bta-miR-147 were significantly up-regulated in IL and ILLN in the JDP cows and IL of MAPT cows compared to HC (only bta-miR-146a was up-regulated in the ILLN in MAPT groups) have associations with bacterial infections (*Mycobacterium bovis* infection, metritis and mastitis) in cattle and were consistently up-regulated during the infection processes^108–110^ or involved in inflammation and various human diseases^111^. Meanwhile, miR-146b was found to assist or cooperate with miR-146a to regulate immune response in humans^112,113^. Because of their association with inflammation, bta-miR-146a and bta-miR-146b were selected as potential biomarkers of bovine mastitis^114,115^. Furthermore, bta-miR-146a was highly expressed as well as DE in ILLN, and miR-146a has been associated with lymph node metastasis and receptor status in human lymphatic diseases^116,117^. MiR-147 was reported to be negatively induced by toll-like receptors stimulation and to regulate inflammatory responses in murine macrophage and human diseases^111,118^. Being DE in both tissues suggests that bta-miR-146a, bta-miR-146b, and bta-miR-147 may play important roles in the MAP infection process and have potential as biomarkers for the development of management strategies of JD. Bta-miR-2305 was significantly DE in all three comparisons in the ILLN. This miRNA has been found to be involved in nacre formation (similar to bone formation) in pearl oyster *Pinctada martensii*^119^.

Considering the JDP and MAPT phenotypes, it is clear from the DE results that mostly different sets of miRNAs were regulating the host response to JDP and MAPT phenotypes in both tissues. For example, out of the 17 and 8 miRNAs DE in the IL for the JDP vs. HC and MAPT vs. HC comparisons, respectively, only 5 (bta-miR-146a, bta-miR-125a, bta-miR-146b, bta-miR-147 and bta-miR-193b) were common and had the same direction of expression but with higher fold upregulation or downregulation in the JDP vs. HC comparison. Moreover six DE miRNAs identified for JDP vs. MAPT in the IL included four (novelmiR_22_13887295, bta-miR-370, bta-miR-129 and bta-miR-129-5p) that were also identified as DE in JDP vs. HC, and showed significantly down-regulated expression in MAPT cows, but similar expression levels in MAPT and HC cows. Meanwhile in the ILLN, 20 and 21 DE miRNAs were identified for the JDP vs. HC and MAPT vs. HC comparisons, respectively out of which only 3 DE miRNAs (bta-miR-146a, bta-miR-21-5p and bta-miR-2305) were common to both comparisons. Notable, eight out of 15 DE mRNAs identified for ILLN JDP vs. MAPT comparison were also identified as DE in JDP vs. HC, while two were identified as DE between MAPT vs. HC cows. Bta-miR-2305 was identified as DE in three comparisons, showing highest expression in JDP but lowest expression in MAPT. Three (bta-miR-375, bta-miR-2285e and bta-miR-146b) and four (bta-miR-99a-5p, novelmiR_23_14116595, bta-miR-100, and bta-miR-452) DE miRNAs showed highest and lowest expression levels in JDP than MAPT and HC, but their expressions were not significantly different between MAPT and HC. These results suggest that the JDP and MAPT phenotypes were largely regulated by different sets of DE miRNAs, that miRNAs may play important roles in the transition from JDP (MAP positive cows) to MAPT phenotype, and that the mechanisms that cows use to develop tolerance to MAP infection include miRNA regulation.

Studies suggest that miRNAs could play a crucial role in modulating the liver’s response to chronic infections^120,121^. Given that MAP affects the immune system and induces inflammation, miRNAs might regulate key inflammatory signaling pathways, liver fibrosis, and metabolic processes in response to such infections. However, in this study liver tissues were missing for several cows which explains why only three DE miRNAs (bta-miR-2483-3p, bta-miR-103 and bta-miR-2305) were identified in the liver. Out of these, bta-miR-103 has been identified as a biomarker for Alzheimer disease risk and progression^122^, indicating potential roles during MAP infection.

As expected, the immune-related GO terms and pathways enriched for target genes of DE miRNAs in IL and ILLN suggest important roles of DE miRNAs in the regulation of the immune response to MAP. IL DE miRNAs of JDP and MAPT groups compared to HC were significantly enriched in GO terms and KEGG pathways related to the immune response and disease. For instance, GO terms related to activities of immune-related cells, such as, regulation of leukocyte mediated immunity, lymphocyte migration, leukocyte chemotaxis, cytokine receptor activity, and positive regulation of interleukin-4 production are known to play important roles in the development of humoral and cellular immunity^123,124^. Similarly, pathways involved in the immune response such as Chemokine signaling pathway, B cell receptor signaling pathway, T cell receptor signaling pathway, Viral protein interaction with cytokine and cytokine receptor, and C-type lectin receptor signaling pathway, etc., significantly enriched in the IL supports regulatory roles of implicated miRNAs of immune processes in the IL. Involvement of a number of these pathways in MAP infection in bovine has been reported^39,41,45,102,125–128^ again supporting regulatory roles of the implicated DE miRNAs in MAP pathogenesis in the IL. Meanwhile, the DE miRNAs identified in the ILLN were significantly enriched in fewer GO terms or pathways related to immune functions, which is supported by data on DNA methylation alterations in the same samples^102^. The immune-related pathways enriched for by target genes of DE miRNAs in ILLN of JDP group (Chemokine signaling pathway, Positive regulation of interleukin-8 production and Positive regulation of type II interferon production) and MAPT group (Positive regulation of chemotaxis and Positive regulation of leukocyte chemotaxis) suggest immune regulatory roles during MAP infection. Some of these pathways have been associated with MAP infection and other diseases of livestock^45^.

The target genes of DE miRNAs of IL and ILLN (JDP/MAPT groups compared to HC) were significantly enriched in processes and functional pathways related to development and growth. The enrichment of biological processes such as regulation of heart contraction, blood circulation, vascular processes in the circulatory system, and cardiac muscle cell contraction by target genes of DE miRNAs in IL and ILLN of JDP/MAPT cows compared to controls indicate involvement in the regulation of heart functions and the circulatory system (Table S6). Chronic infections like MAP can cause systemic inflammation, which may affect heart function and blood circulation. The body’s response to infection often involves significant changes in blood flow and pressure. The immune system’s response to JD involves various cells and molecules that travel through the circulatory system. Changes in vascular processes could reflect increased immune cell trafficking and inflammation. Additionally, the DE miRNAs in the IL of JDP cows compared to HC were significantly enriched in the superoxide metabolic process and reactive oxygen species (ROS) metabolic process. MAP infection progressively leads to chronic inflammation, producing ROS as a byproduct. This oxidative stress can damage tissues and disrupt normal cellular functions, leading to changes in metabolic processes, including those related to heart function. Superoxide, a type of ROS, is crucial in managing oxidative stress. The observation of these metabolic pathways suggests a response to increased oxidative damage in MAP-infected cows (JDP). Moreover, the DE miRNAs identified in both the IL and ILLN of JDP/MAPT cows compared to HC were significantly enriched in the glucan metabolic process. JD cows may experience metabolic shifts as their bodies try to cope with the infection. Changes in carbohydrate metabolism could reflect an adaptation to altered energy needs or availability. Glucans, which are polysaccharides, play a role in immune responses, and changes in their metabolism could be linked to the body’s effort to fight the infection. The enrichment of DE miRNAs in IL and ILLN in these diverse biological and functional processes suggests the important regulatory roles of DE miRNAs during JD. These findings provide insights into the regulatory network underlying the complex phenotype of JD. This is supported by a recent findings that MAP exploits miRNA to modulate host metabolism during infection^11,60^.

The targets of DE miRNAs identified in ILLN between JDP vs. MAPT cows were enriched in more GO terms and KEGG pathways, indicating the multifaceted roles of DE miRNAs across immune modulation, muscle and heart function, metabolism, and cellular signaling. Firstly, a number of these DE miRNAs appear to be involved in immune regulation and inflammation. Notably, enrichment in pathways such as the Notch signaling pathway, JAK-STAT signaling, and Leukocyte transendothelial migration suggest that these miRNAs may modulate critical immune processes, including leukocyte trafficking and immune cell differentiation. This could influence the host’s ability to either mount an effective response or tolerate chronic infection with minimal clinical manifestations. Additionally, the enrichment in the TGF-beta signaling pathway and the negative regulation of wound healing further implicates these miRNAs in controlling immune responses and tissue remodeling during chronic MAP infection, potentially regulating the balance between pro-inflammatory and anti-inflammatory signals. Secondly, several enriched terms related to muscle structure and function were observed, such as cardiac muscle cell development, myofibril assembly, and regulation of heart contraction. These suggest a possible role for miRNAs in maintaining muscle integrity and function during chronic infection, which may be crucial given the potential systemic effects of MAP on infected animals. Furthermore, metabolic regulation emerged as a key area of involvement, with enrichment in terms like ATP transmembrane transporter activity, carbohydrate derivative transmembrane transporter activity, and the pentose phosphate pathway. These findings suggest that miRNAs could regulate energy homeostasis and nutrient transport in the liver, which may be particularly relevant under the chronic inflammatory conditions associated with MAP infection. This metabolic regulation may reflect a host adaptation aimed at sustaining energy levels and balancing metabolic needs during prolonged infection. In conclusion, these findings indicate that the DE miRNAs in ILLN between JDP and MAPT cows are likely involved in complex biological processes, including immune modulation, muscle function, metabolic regulation, and cellular signaling. These roles may collectively contribute to the differential response to MAP infection, where tolerant cows are able to harbor the pathogen without developing clinical disease or spreading the infection.

Interestingly and although most enriched GO terms and KEGG pathways were unique to individual comparisons (Fig. 5), a subset of terms were commonly enriched across multiple comparisons, suggesting unique biological mechanisms driving the JDP and MAPT phenotypes as well as shared biological activities underlying MAP infection. Notably, immune-related processes such as receptor-mediated endocytosis, cytokine receptor activity, and the viral protein interaction with cytokine and cytokine receptor pathway were consistently enriched across comparisons involving both JDP and MAPT groups in IL, indicating a convergent immune response to MAP despite phenotypic differences. Similarly, terms related to heart contraction and regulation of vascular functions, observed in both IL and ILLN, suggest that systemic physiological responses, possibly triggered by chronic inflammation and oxidative stress, are common features of MAP infection across tissues. These shared pathways highlight core regulatory roles of miRNAs in immune and cardiovascular responses and support the notion that both tolerant (MAPT) and infected (JDP) cows exhibit overlapping molecular alterations, particularly in pathways essential for host defense and systemic adaptation to infection. Most importantly, our data clearly shows that different biological processes and pathways were driving the JDP and MAPT phenotypes.

## Conclusion

The repertoire of miRNAs identified in this study (441 in the IL, 405 in the ILLN, and 201 in liver, including 129, 103, and 21 novel miRNAs, respectively) will enrich the bovine miRNA catalogue and enhance our knowledge of the roles of miRNAs in JD. The study demonstrated potential tissue-specific regulatory roles of miRNAs in response to MAP infection, revealing diverse DE miRNAs, crucial biological processes and functional pathways among JDP, MAPT and HC cows in IL and ILLN. Notably, some of the miRNAs DE in both IL and ILLN tissues (bta-miR-146b, bta-miR-375, bta-miR-21-5p, bta-miR-146a, bta-miR-125a, bta-miR-100, bta-miR-147, bta-miR-99a-5p, bta-miR-320, bta-miR-146a, bta-let-7b) have known correlations with bovine bacterial infections, highlighting their potential as biomarkers for MAP infection. Finally, the results of this study underscore the significant regulatory roles of miRNAs in the IL and ILLN in mediating both immune and systemic responses to MAP infection and also that mainly different sets of DE miRNAs, biological processes and pathways were driving the JDP and MAPT phenotypes. However, further functional research is required to explore the specific roles of the identified DE miRNAs in response to MAP infection.

## Supplementary Information

Below is the link to the electronic supplementary material.

Supplementary Material 1

Supplementary Material 2

Supplementary Material 3

Supplementary Material 4

Supplementary Material 5

Supplementary Material 6

Supplementary Material 7

## Data Availability

The sequence data generated and that support the findings of this study have been deposited in the National Center for Biotechnology and Information (NCBI) Sequence Read Archive (SRA) under the BioProject PRJNA1255591 and data generated are submitted as supplementary files.

## Declarations

### ARRIVE guidelines

I confirm that this study is reported in accordance with ARRIVE guidelines.
