# Supplementary material for: MicroRNA-mediated regulatory mechanisms in cow’s ileum and ileum lymph node in response to Mycobacterium avium subspecies paratuberculosis infection
Source: Sci Rep. 2025 Dec 3;15:43030. doi: 10.1038/s41598-025-23969-x (PMC12675627; doi:10.1038/s41598-025-23969-x)
Supplement: Supplementary file 1 — Supplementary Material 1 [file 41598_2025_23969_MOESM1_ESM.pdf]

d / Non classifié

A- Ileum

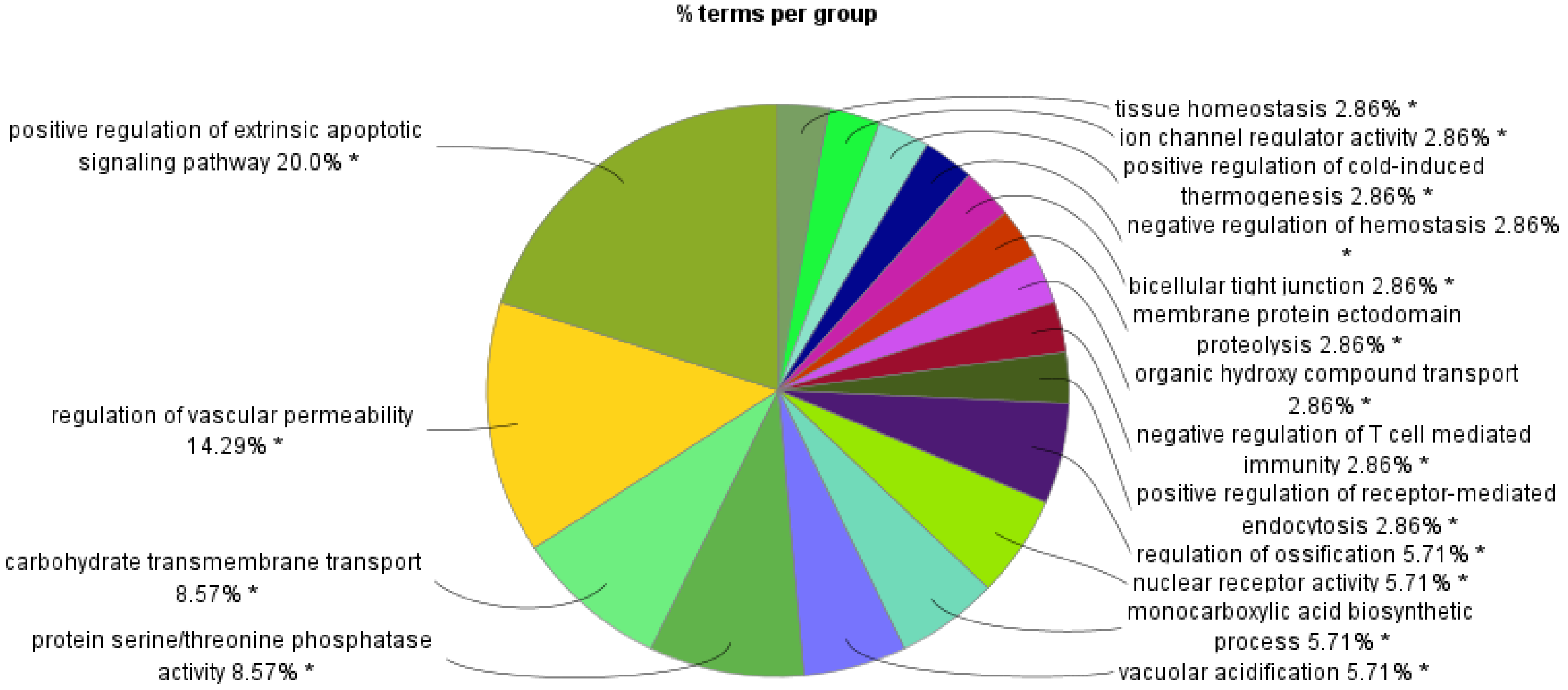

B- Ileal lymph node

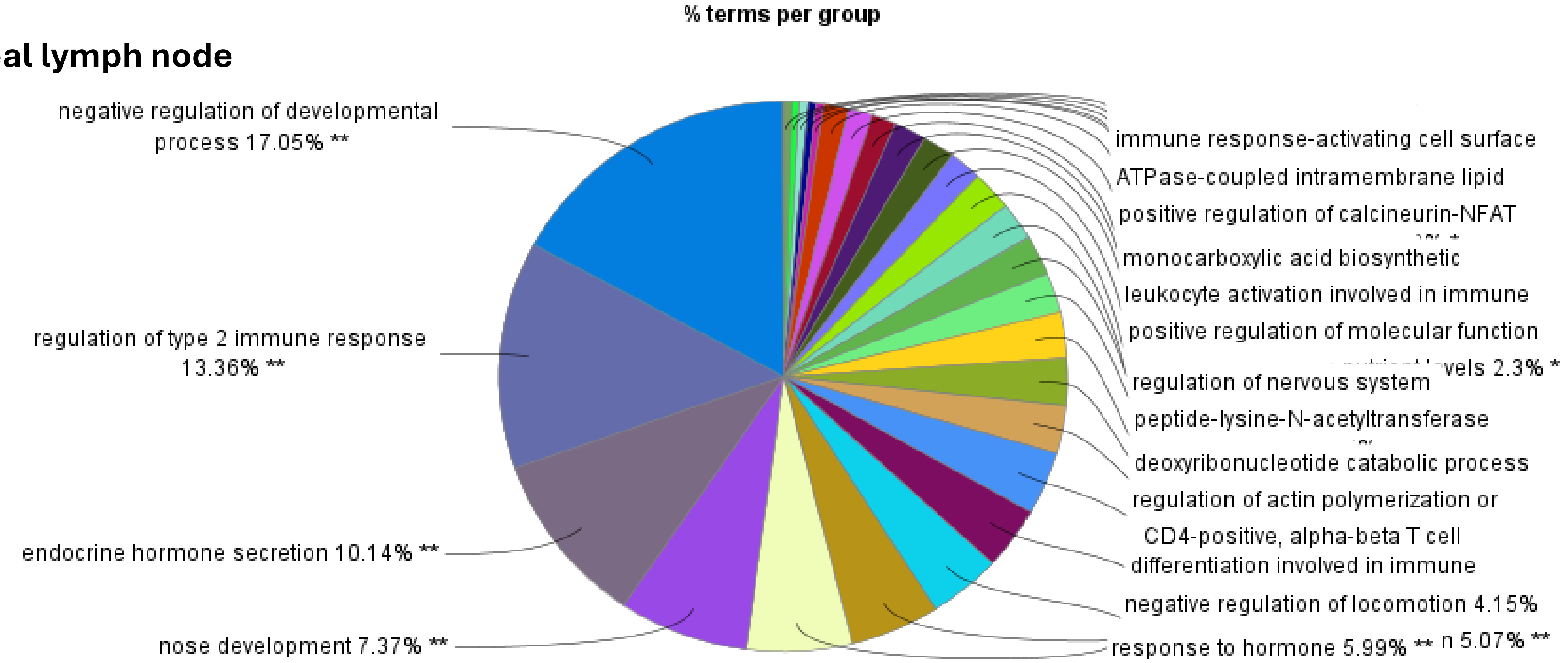

Figure S1: Functional terms (gene ontology and pathways) enriched by target genes of highly expressed miRNAs in the ileum clustered into 17 functional groups (A) and in the ileal lymph node into 27 functional groups (B)
